# Ability of procalcitonin to distinguish between bacterial and nonbacterial infection in severe acute exacerbation of chronic obstructive pulmonary syndrome in the ICU

**DOI:** 10.1186/s13613-021-00816-6

**Published:** 2021-03-06

**Authors:** Cédric Daubin, François Fournel, Fabrice Thiollière, Fabrice Daviaud, Michel Ramakers, Andréa Polito, Bernard Flocard, Xavier Valette, Damien Du Cheyron, Nicolas Terzi, Muriel Fartoukh, Stephane Allouche, Jean-Jacques Parienti, from the PROCALCIVIR and BPCTrea study group

**Affiliations:** 1 Department of Medical Intensive Care, CHU de Caen, 14000 Caen, France; 2 Department of Biostatistics and Clinical Research, CHU de Caen, 14000 Caen, France; 3 Intensive Care Unit, Centre Hospitalier Lyon Sud, Pierre Bénite, Hospices Civils de Lyon, France; 4 Department of Medial Intensive Care, Cochin University Hospital, Paris, France; 5 Department of Intensive Care Medicine, General Hospital, Saint Lô, France; 6 Service de Médecine Intensive Et Réanimation, General Intensive Care Unit, Hôpital Raymond Poincaré (APHP), Raymond Poincaré Hospital, Garches, France; 7 Laboratoire Infection & Inflammation, U1173 Université de Versailles SQY–Paris Saclay - INSERM, Garches, France; 8 Department of Anesthesiology and Critical Care Medicine, Edouard Herriot Hospital, Hospices Civils de Lyon, Lyon, France; 9 Department of Medical Intensive Care, CHU de Grenoble Alpes, 38000 Grenoble, France; 10 INSERM, U1042, University of Grenoble-Alpes, HP2, 38000 Grenoble, France; 11 Service de Medecine Intensive Reanimation, AP-HP, Sorbonne université, Hôpital Tenon, Groupe de Recherche Clinique CARMAS, collegium Gallilée, Paris, France; 12 Universite Caen Normandie, Medical School, EA 4650, Signalisation, Electrophysiologie et Imagerie des lésions d’Ischemie-reperfusion Myocardique, 14000 Caen, France; 13 EA2656 Groupe de Recherche sur l’Adaptation Microbienne (GRAM 2.0), Université Caen Normandie, Caen, France

**Keywords:** Chronic obstructive pulmonary disease, Procalcitonin, Antibiotic stewardship, Respiratory tract infection, Community-acquired pneumonia, Viral infection

## Abstract

**Background:**

To assess the ability of procalcitonin (PCT) to distinguish between bacterial and nonbacterial causes of patients with severe acute exacerbation of COPD (AECOPD) admitted to the ICU, we conducted a retrospective analysis of two prospective studies including 375 patients with severe AECOPD with suspected lower respiratory tract infections. PCT levels were sequentially assessed at the time of inclusion, 6 h after and at day 1, using a sensitive immunoassay. The patients were classified according to the presence of a documented bacterial infection (including bacterial and viral coinfection) (BAC + group), or the absence of a documented bacterial infection (i.e., a documented viral infection alone or absence of a documented pathogen) (BAC- group). The accuracy of PCT levels in predicting bacterial infection (BAC + group) vs no bacterial infection (BAC- group) at different time points was evaluated by receiver operating characteristic (ROC) analysis.

**Results:**

Regarding the entire cohort (*n* = 375), at any time, the PCT levels significantly differed between groups (Kruskal–Wallis test, *p* < 0.001). A pairwise comparison showed that PCT levels were significantly higher in patients with bacterial infection (*n* = 94) than in patients without documented pathogens (*n = *218) (*p* < 0.001). No significant difference was observed between patients with bacterial and viral infection (*n* = 63). For example, the median PCT-H_0_ levels were 0.64 ng/ml [0.22–0.87] in the bacterial group vs 0.24 ng/ml [0.15–0.37] in the viral group and 0.16 ng/mL [0.11–0.22] in the group without documented pathogens. With a c-index of 0.64 (95% CI; 0.58–0.71) at H_0_, 0.64 [95% CI 0.57–0.70] at H_6_ and 0.63 (95% CI; 0.56–0.69) at H_24_, PCT had a low accuracy for predicting bacterial infection (BAC + group).

**Conclusion:**

Despite higher PCT levels in severe AECOPD caused by bacterial infection, PCT had a poor accuracy to distinguish between bacterial and nonbacterial infection. Procalcitonin might not be sufficient as a standalone marker for initiating antibiotic treatment in this setting.

## Background

Procalcitonin (PCT) is considered useful for determining the likelihood that patients will develop bacterial infections, and several large randomized controlled clinical trials have investigated the ability of a PCT-based strategy to safely reduce antibiotic exposure in noncritically ill patients with lower respiratory tract infections [1–7]. Interestingly, two recent meta-analyses [8, 9], focusing on PCT-guided antibiotics in a mixed population of AECOPD (i.e., in the ICU and not in the ICU), suggested that a PCT-guided antibiotic strategy reduced antibiotic prescriptions compared with standard management without affecting clinical outcomes such as treatment failure, length of hospitalization and rate of re-exacerbation or overall mortality. These results suggested that PCT could be a useful biomarker to guide antibiotic therapy in this setting. However, due to the methodological limitations and the small overall study population, the authors underlined that the quality of the available evidence was considered low to moderate.

In addition, the safety of PCT-based strategies in critically ill medical patients is not clear. Despite PCT-based algorithms appear safe and reduce antibiotic exposure in critically ill patients [10–18], only few trials [11, 12] were designed to assess the impact on mortality. These studies reported contradictory results. In addition, in critically ill patients, the results of different meta-analyses assessing the effect of PCT-guided therapy on mortality are inconsistent [19–22].

Recently, a large multicenter randomized controlled clinical study from our group among AECOPD patients admitted in ICU showed that the use of PCT was not non-inferior to standard of care regarding 3-month mortality. Moreover, the PCT-guided strategy was significantly worse for patients not on antibiotics at baseline [23].

To explore this disappointing result, we hypothesized that PCT could fail to distinguish between bacterial and nonbacterial causes of severe AECOPD. Therefore, we aimed to assess the ability of PCT to distinguish between bacterial and nonbacterial causes of severe AECOPD, using all the data sets we previously published [23–25].

## Methods

### Patients

We conducted a retrospective analysis from two data sets of prospective studies (i.e., the PROCALCIVIR study, an observational cohort [24, 25] and the BPCTrea study, a randomized controlled trial [23]) including patients with severe AECOPD with suspected lower respiratory tract infections (detailed information provided in the Additional file 1: Appendix). The detailed study designs and main results have been published previously [23–25]. Briefly, a PROCALCIVIR study was conducted between September 2005 and September 2006 in one ICU in France. The BPCTrea study was conducted between October 2010 and March 2016 in 11 ICUs in France. PROCALCIVIR and BPCTrea studies allowed for antibiotics at the time of inclusion. These studies were conducted according to the principles of the Declaration of Helsinki.

### Procedures

Circulating PCT levels were sequentially assessed at inclusion (PCT-H_0_), at 6 h after inclusion (PCT-H_6_), and on day 1 after inclusion (PCT-H_24_). PCT levels were measured by Elecsys BRAHMS PCT immunoassay (Roche Diagnostics GmbH, Mannheim, Germany) using a Cobas e411 analyzer, according to the manufacturer’s instructions.

In both, the PROCALCIVIR and BPCTrea studies, the microbiological investigation was encouraged, but left to the discretion of the attending physicians, according to the usual practice in each center.

Patients were classified according to the presence of a documented bacterial infection (including bacterial and viral coinfection) (BAC + group), or absence of a documented bacterial infection (i.e., a documented viral infection alone or absence of documented pathogens) (BAC− group).

### Statistical analysis

Data are expressed as the mean ± SD or median (interquartile range [IQR]) and percentage, depending on the type of variable of interest. PCT levels were compared between the three groups (i,e., documented bacterial infection, documented viral infection alone and group without documented pathogens) using the Kruskal–Wallis test. In addition, a pairwise comparison between groups was performed using the Wilcoxon test. The discriminative ability of PCT in predicting documented bacterial infection (BAC +) vs nondocumented bacterial infection (BAC−) was evaluated by the receiver operating characteristic (ROC) analysis at each time point (i.e., H0, H6 and H24 after inclusion) in the overall cohort and in predefined subgroups (i.e., AECOPD with and without pneumonia and patients with or without antibiotics at inclusion). The area under the curve (AUC) and 95% confidence intervals are provided. Prism 8.3.1 (GraphPad software, LLC, San Diego, USA) was used for the data analysis. All tests were 2-sided, and a *p*-value < 0.05 was considered statistically significant.

## Results

### Patient characteristics

The baseline characteristics of the two studied cohorts are shown Table 1. Of 375 included patients, 94 (25%) patients had a documented bacterial infection (BAC + group) (including 76 (20%) patients with bacterial infection alone and 18 (5%) patients with bacterial and viral coinfection). Two hundred eighty one patients (75%) had nondocumented bacterial infection (BAC− group) (including 63 (17%) patients with documented viral infection alone and 218 patients without documented pathogens). Overall, 159 patients had severe AECOPD with pneumonia, and 202 patients received antibiotics at the time of inclusion.

**Table 1:** Patient characteristics

	BPCTrea patients *N* = 302		Procalcivir patients *N* = 73	
	Without pneumonia *N* = 177	With pneumonia *N* = 125	Without pneumonia *N* = 39	With pneumonia *N* = 34
Age (years), mean ± SD	67 ± 10	70 ± 11	62 ± 15	70 ± 10
Men n (%)	126 (71)	82 (66)	26 (67)	28 (82)
Current smoker n (%)	76 (43)	38 (30)	11 (28)	11 (32)
Comorbidities				
Arterial hypertension n (%)	83 (47)	73 (58)	15 (38)	17 (50)
Cardiopathy n (%)	61 (34)	44 (35)	8 (15)	23 (67)
Diabetes mellitus n (%)	30 (17)	29 (23)	13 (33)	13 (38)
Severity of COPD n (%)				
GOLD stage 0–I	15 (8)	9 (7)	7 (18)	2 (6)
GOLD stage II	18 (10)	29 (23)	3 (8)	9 (26)
GOLD stage III	58 (33)	33 (26)	3 (8)	3 (9)
GOLD stage IV	69 (39)	42 (34)	26 (67)	20 (59)
GOLD stage unknown	12 (7)	17 (14)	–	–
Home oxygen n (%)	74 (42)	53 (42)	22 (56)	18 (53)
Home noninvasive ventilatory support n (%)	42 (24)	32 (25)	5 (13)	6 (18)
Severity of illness				
SAPS II, median (Q1–Q3)	32 (26-41)	38 (30-46)	30 (23–35)	37 (20–50)
Pneumonia severity index class n (%)				
I–III		43 (34)		4 (11)
IV		15 (12)		14 (42)
V		67 (54)		16 (47)
Mechanical ventilation at the time of inclusion n (%)				
Invasive n (%)	45 (25)	37 (30)	6 (15)	14 (41)
Noninvasive n (%)	108 (61)	74 (60)	25 (64)	20 (58)
Antibiotics at the time of inclusion n (%)	93 (53)	89 (71)	9 (23)	11 (32)
Documented infection n (%)				
Bacterial	36 (20)	38 (30)	5 (13)	15 (44)
Viral	38 (21)	28 (22)	9 (23)	5 (15)
PCT H0 (μg/L), median ((Q1-Q3)	0.16 (0.08–0.49)	0.42 (0.14–0.86)	0.10 (0.07–0.18)	0.49 (0.13–1.47)

### Microbiological findings

Lower respiratory samples were taken from 268 (71%) patients, including sputum (*n* = 176), tracheobronchial aspirate (*n* = 35), bronchoalveolar lavage (*n* = 12), and distal protected specimens (*n* = 73). In addition, blood culture was performed in 93 (25%) patients. Additionally, serological diagnosis for antibodies to *Legionella pneumophila* or detection of *Legionella pneumophila* serogroup 1 urinary antigen test was performed in 297 (79%) patients. A polymerase chain reaction (PCR) assay for viral study was performed in 258 (69%) patients. The test included the following viruses: V. influenza A/H1/H3/H1N1v/B, parainfluenza 1, 2, 3, 4, VRS A/B, metapneumovirus, entero-rhinovirus, coronavirus OC43/229E/NL63/HKU1/MERS, adenovirus and bocavirus. *Mycoplasma pneumoniae, Legionella pneumophila, Bordetella pertussis, and Chlamydophila pneumoniae* were also detected by PCR assay. The most frequently isolated bacteria were *Haemophilus influenza* (*n* = 23), *Pseudomonas spp.* (*n* = 16), *Streptococcus pneumonia* (*n* = 8), *Streptococcus spp. (n* = *6) and Staphylococcus aureus (n* = *6)*. The most frequently isolated viruses were rhinovirus (n = 23), syncytial respiratory virus (*n* = 16), parainfluenzae virus (*n* = 10), coronavirus (*n* = 9) and influenzae A/B virus (*n* = 9/1).

### PCT levels

**Fig. 1:**
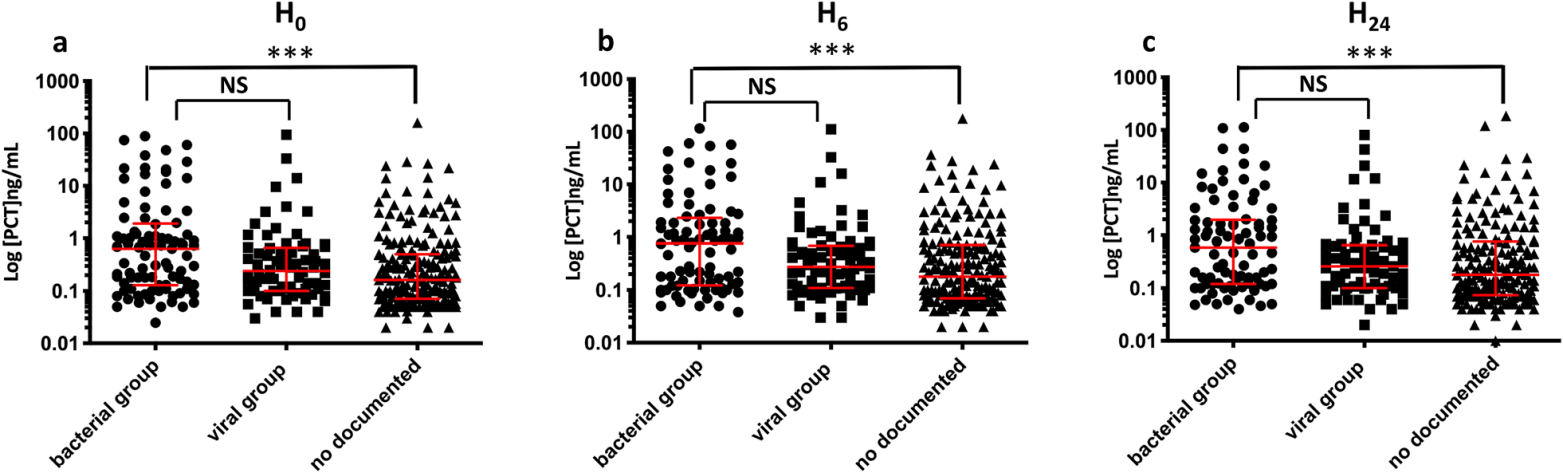
PCT levels at inclusion (PCT-H_0_) (panel a), at 6 hours (PCT-H_6_) (panel b) and day 1 (PCT-H_24_) (panel c) after inclusion, in accordance with the documented infection group ***p < 0.001

The circulating PCT levels were sequentially assessed at inclusion (PCT-H_0_) in 355 (95%) patients, at six hours after inclusion (PCT-H_6_) in 341 (91%) patients, and on day 1 after inclusion (PCT-H_24_) in 331 (89%) patients. The PCT levels of the documented infection group are shown Fig. 1a–c. At any time, PCT levels significantly differed between groups (Kruskal–Wallis test, *p* < 0.001). Using a Wilcoxon test, the PCT levels were significantly higher among patients with bacterial infection (*n* = 94) than among patients without documented pathogens (*n* = 218) (*p* < 0.001) but no significant difference was observed between patients with bacterial infection and patients with viral infection (*n* = 63). The median PCT-H_0_ levels were 0.64 ng/mL [0.22–0.87] in the bacterial group vs 0.24 ng/mL [0.15–0.37] in the viral group and 0.16 ng/mL [0.11–0.22] in the group without documented pathogens. PCT-H_6_ levels were 0.76 ng/mL [0.22–1.11] in the bacterial group vs 0.27 ng/mL [0.20–0.45] in the viral group and 0.18 ng/mL [0.12–0.27] in the group without documented pathogens. PCT-H_24_ levels were 0.59 ng/mL [0.20–1.17] in the bacterial group vs 0.26 ng/mL [0.16–0.40] in the viral group and 0.18 ng/mL [0.13–0.26] in the group without documented pathogens.

We conducted subgroup analyses among patients with (ATB^+^) and without (ATB^−^) antibiotics and with (PNP^+^) or without (PNP^−^) pneumonia at the time of inclusion. In the subgroup of patients with antibiotics, PCT levels significantly differed between groups. PCT levels were significantly higher in patients with documented bacterial infection compared to the other groups (detailed information regarding each group may be found in the Additional file 1: Appendix). No difference in PCT levels was observed in the subgroup of patients without antibiotics at the time of inclusion (detailed information regarding each group may be found in the Additional file 1: Appendix). In the subgroup with pneumonia, we observed no difference in PCT levels between the different groups (detailed information regarding each group may be found in the Additional file 1: Appendix). In contrast, in the subgroup of patients without pneumonia, PCT levels significantly differed between groups. PCT levels were significantly higher in patients with documented infection (i.e., bacterial and viral groups) compared to the group without documented pathogens (detailed information regarding each group may be found in the Additional file 1: Appendix).

### Ability of PCT to distinguish between bacterial (BAC + group) and nonbacterial infections (BAC−group)

**Fig. 2:**
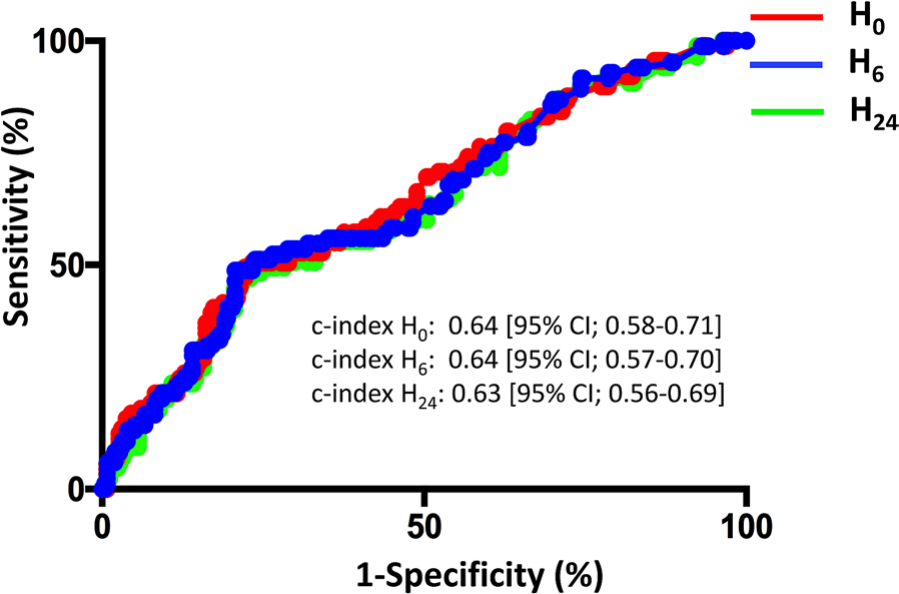
Receiver operating characteristic (ROC) curves at any time (i.e., H_0_, H_6_ and H_24_ after inclusion) for the prediction of documented bacterial infection (including bacterial and viral coinfection) (BAC + group) vs nondocumented bacterial infection (i.e., documented viral infection alone or absence of documented pathogen) (BAC− group) for the PCT levels

The ROC curves at any time (i.e., H_0_, H_6_ and H_24_ after inclusion) for the prediction of documented bacterial infection (including bacterial and viral coinfection) (BAC + group) vs nondocumented bacterial infection (i.e., documented viral infection alone or absence documented pathogens) (BAC− group) for the PCT levels are shown Fig. 2. With a c-index of 0.64 (95% CI 0.58–0.71) at H_0_, 0.64 [95% CI 0.57–0.70] at H_6_ and 0.63 (95% CI 0.56–0.69) at H_24_, PCT had a low accuracy for predicting bacterial infection.

Similar results were observed in subgroups of patients with (ATB^+^) and without (ATB^−^) antibiotics and with (PNP^+^) or without (PNP^−^) pneumonia at the time of inclusion (detailed information regarding each group may be found in the Additional file 1: Appendix).

## Discussion

In this large cohort of patients with severe AECOPD admitted to the ICU, PCT had a low ability to discriminate those patients with and without bacterial documented infection in the overall cohort and in all predefined subgroups (i.e., patients with and without pneumonia and patients with or without antibiotics at the time of inclusion). This poor performance of PCT may contribute to explain the excess mortality observed in the subgroup of patients without antibiotics at baseline in the BPCTrea trial [23]. This result suggests that a PCT-based strategy to initiate antibiotic treatment should be considered with cautious in this setting.

To our knowledge, we provided the largest cohort ever assembled investigating the ability of PCT to predict bacterial infection in a homogenous population of critically ill patients with severe AECOPD and suspected lower respiratory tract infection who need noninvasive or invasive mechanical ventilation. PCT failed to predict bacterial infection in the overall cohort and all predefined subgroups. This result contrasts with previous meta-analyses reporting the good diagnostic accuracy of PCT among mixed patients hospitalized for suspected bacterial infections (including patients admitted to the ICU) both to differentiate bacterial infections from viral infections and to differentiate bacterial infections from other noninfective causes of systemic inflammation [26–29]. However, although PCT could be considered an accurate marker for diagnosing bacterial infection, the result of this test needs to be interpreted with caution and might not be sufficient as a standalone marker for initiating antibiotic treatment, specifically in ICU patients [27, 30]. This result is supported by a recent prospective multicenter trial in ICU patients addressing a large panel of circulating biomarkers previously tested in a sepsis setting to differentiate sepsis from nonseptic SIRS [31]. In that study [31], no biomarker, alone or in combination, was able to detect infection. With a ROC-AUC of 0.55 [0.47–0.62], PCT poorly discriminated sepsis from nonseptic SIRS [31], a result consistent with those reported here. In addition, a recent meta-analysis [8] focusing on the potential of PCT in predicting bacterial exacerbation in severe COPD exacerbation, reported an area under the receiver operator characteristic curve of 0.77 [0.73–0.80], indicating moderate accuracy. A subgroup analysis revealed that the pooled sensitivity and specificity of PCT for patients admitted in ICU was lower than for other patients.

Algorithms using PCT in critically ill patients suspected of bacterial infection can lead to two opposite clinical decisions: (i) initiate antibiotics; (ii) discontinue antibiotics. Today, a prompt empirical antibiotherapy remains strongly recommended in critical patients suspected of sepsis. The use of procalcitonin algorithms may expose to the risk to delay antibiotic treatment and therefore to increase mortality. Despite previous randomized trials [10–18] reporting that PCT-based algorithm appears safe and reduces antibiotic exposure in critically ill patients, trials specially designed to demonstrate the noninferiority of PCT-guided strategies with respect to mortality in the ICU are scarce [11, 12, 23] and inconsistent. Differences in the PCT-guided antibiotic algorithms may explain this heterogeneity. In procalcitonin algorithms providing rules for the initiation, continuation and discontinuation of antibiotic treatment [11, 23], one study [11] reported that 60-day mortality was non-inferior in the experimental group (non-inferiority margin of 10% almost reached) compared to the standard of care group, while our group [23] failed to demonstrate noninferiority with respect to 3-month mortality **(**non-inferiority margin of 12% exceeded). In one study [12] focusing only on the de-escalation of antibiotic therapy, 28-day and 1-year mortality were significantly lower in the PCT group than in the control group and the non-inferiority margin was 8%. These results suggest that PCT might be more useful for stopping than initiating antibiotics in critically ill patients.

Our large pooled data analysis supports the recommendation that PCT alone should not replace clinical decision for antibiotic initiation, in particular among severe AECOPD. In addition, any delay in antibiotic prescription in such situation could lead to poorer outcome. In this line, a Cochrane meta-analysis [32] concluded that antibiotics reduced the risk of treatment failure in patients with severe AECOPD hospitalized in the ICU and reduced mortality. One explanation could be that antibiotics prevent the risk of bacterial infection due to an impaired phagocytosis of alveolar macrophage in COPD patient. Interestingly, the impairment of phagocytosis could be both mediated by virus infections such as human rhinovirus and related to disease severity [33]. Nevertheless, further studies are needed to assess the usefulness and safety of PCT to withhold or stop antibiotics in critically ill AECOPD patients.

Several limitations of the study warrant discussion. First, this study was a retrospective analysis of two different prospective cohorts [23–25]. However, a separate analysis of each cohort showed similar results (data not shown) indicating homogenous data among the two studies. Second, we chose to include in the BAC + group all patients in whom a positive bacterial culture of respiratory tract samples was observed. However, there is no absolute “gold standard” for the diagnosis of bacterial infection in AECOPD since a bacterial colonization is frequently detected. Therefore, a risk of misclassification is not totally excluded. This limitation is not specific to our study. Third, despite a large microbiological investigation, we reported a high percentage of patients without documented pathogen. This finding might be explained by a relatively high proportion of patients with antibiotic at ICU inclusion. In contrast, the proportion of positive PCR results is consistent with recent large cohort of AECOPD requiring hospital admission [34]. Fourth, considering the number of documented bacterial and viral infection, we cannot exclude a lack of power of the study. Fifth, antibiotic treatment before the inclusion of some patients may have affected PCT levels. However, previous reports showed that PCT levels were similar among patients pretreated and not pretreated with antibiotics [3, 4, 23–25]. Moreover, higher PCT values were observed for the subgroup of patients with antibiotics in the documented bacterial group (see the Additional file 1: Appendix).

## Conclusion

In this study, PCT predicted bacterial infection with poor accuracy in patients with severe AECOPDs admitted in ICU. Therefore, a PCT-based strategy to initiate antibiotic treatment should be considered with cautious in this setting. Further studies are needed to assess the usefulness and safety of PCT to withhold or stop antibiotics in critically ill AECOPD patients.

## Supplementary Information

**Additional file 1: Online Resource 1.** Detailed information regarding inclusion, no inclusion and exclusion criteria and definitions.** Online Resource 2.** PCT levels at inclusion (PCT-H0), at six hours (PCT-H6) and day 1 (PCT-H24) after inclusion, in subgroups of patients with (ATB+) (Panel** a**-**c**) and without (ATB-) (Panel** d**-**f**) antibiotics at inclusion.** Online Resource 3.** PCT levels at inclusion (PCT-H0), at six hours (PCT-H6) and day 1 (PCT-H24) after inclusion, in subgroups of patients with (PNP+) (Panel** a**-**c**) or without (PNP-) (Panel** d**-**f**) pneumonia at inclusion.** Online Resource 4.** Receiver operating characteristic (ROC) curves at any time (i.e., H0, H6 and H24 after inclusion) for the prediction of documented bacterial infection (including bacterial and viral coinfection) vs nondocumented bacterial infection (i.e., documented viral infection alone or absence of documented pathogen) for the PCT levels in subgroups of patients with (ATB+) (Panel** a**-**c**) and without (ATB-) (Panel** d**-**f**) antibiotics at inclusion.** Online Resource 5**. Receiver operating characteristic (ROC) curves at any time (i.e., H0, H6 and H24 after inclusion) for the prediction of documented bacterial infection (including bacterial and viral coinfection) vs nondocumented bacterial infection (i.e., documented viral infection alone or absence of documented pathogen) for the PCT levels in subgroups of patients with (PNP+) (Panel** a**-**c**) or without (PNP-) (Panel** d**-**f**) pneumonia at inclusion.

## Data Availability

The datasets used and/or analyzed during the current study are available from the corresponding author on reasonable request.

## Abbreviations

AECOPD: Acute exacerbation of chronic obstructive pulmonary disease
AUC: Area under the curve
ICU: Intensive care unit
IQR: Interquartile range
PCT: Procalcitonin
ROC: Receiver operating characteristic
