# Supplementary material for: Ability of procalcitonin to distinguish between bacterial and nonbacterial infection in severe acute exacerbation of chronic obstructive pulmonary syndrome in the ICU
Source: Ann Intensive Care. 2021 Mar 6;11:39. doi: 10.1186/s13613-021-00816-6 (PMC7936235; doi:10.1186/s13613-021-00816-6)

**Electronic Supplementary Material**

**Title**

**Ability of procalcitonin to distinguish between bacterial and nonbacterial infection in severe acute exacerbation of chronic obstructive pulmonary syndrome in the ICU**

**Authors**

Cédric Daubin^1,*^, François Fournel^2^, Fabrice Thiollière^3^, Fabrice Daviaud^4^, Michel Ramakers^5^, Andréa Polito^6,7^, Bernard Flocard^8^, Xavier Valette^1^, Damien Du Cheyron^1^, Nicolas Terzi^9,10^, Muriel Fartoukh^11^, Stephane Allouche^12^, Jean-Jacques Parienti^2,13^ from the PROCALCIVIR and BPCTrea study group^†^

^1^Department of Medical Intensive Care, CHU de Caen, Caen, 14000, France

^2^Department of Biostatistics and Clinical Research, CHU de Caen, Caen, 14000, France

^3^Intensive Care Unit, Centre Hospitalier Lyon Sud, Hospices Civils de Lyon, Pierre Bénite, France

^4^Department of Medial Intensive Care, Cochin University Hospital, Paris, France

^5^Department of Intensive Care Medicine, General Hospital, Saint Lô, France

^6^Service de Médecine Intensive et Réanimation, Hôpital Raymond Poincaré (APHP), General Intensive Care Unit, Raymond Poincaré Hospital, Garches, France

^7^Laboratoire Infection & Inflammation, U1173 Université de Versailles SQY–Paris Saclay - INSERM, France U1173 Lab Inflammation & Infection, University of Versailles SQY-Paris Saclay - INSERM, Garches, France

^8^Department of Anesthesiology and Critical Care Medicine, Edouard Herriot Hospital, Hospices Civils de Lyon, Lyon, France

^9^Department of Medical Intensive Care, CHU de Grenoble Alpes, F-38000 Grenoble, France

^10^INSERM, U1042, University of Grenoble-Alpes, HP2, F-38000 Grenoble, France Normandy University, France

^11^Service de Médecine intensive Réanimation, AP-HP. Sorbonne université, Hôpital Tenon; Groupe de Recherche Clinique CARMAS, Collégium Gallilée

^12^Université Caen Normandie, Medical School, EA 4650, Signalisation, Électrophysiologie et Imagerie des Lésions d'Ischémie-reperfusion Myocardique, Caen, F-14000, France Department of Biochemistry, Caen, 14000, France

^13^EA2656 Groupe de Recherche sur l’Adaptation Microbienne (GRAM 2.0), Université Caen Normandie, France

**Members of BPCTrea study group**

Cédric Daubin, Xavier Valette, Amélie Seguin, Jennifer Brunet, Pierre Charbonneau and Damien Du Cheyron (CHU de Caen, Department of Medical Intensive Care, Caen, 14000, France); Stephane Allouche (Department of Biochemistry, Caen, F-14032, France, and Normandie Univ, UNICAEN, CHU Caen, Signalisation, Electrophysiologie et Imagerie des Lésions d’Ischémie-repefusion Myocardique, Caen, F-14032, France CHU de Caen); François Fournel and Jean-Jacques Parienti (CHU de Caen, Department of Biostatistics and Clinical Research, Caen, 14000, France, and EA4655 Risque Microbiens, Caen Normandie Université, Caen, France); Bertrand Sauneuf (Service de Réanimation Médicale Polyvalente, Centre Hospitalier Public du Cotentin, BP 208, 50102 Cherbourg-en-Cotentin, France); Fabrice Thiollière and Julien Bohe (Intensive Care Unit, Hospices Civils de Lyon, Centre Hospitalier Lyon Sud, Pierre Bénite, France); Jean-Paul Mira and Fabrice Daviaud (Department of Medical Intensive Care, Cochin University Hospital, Paris, France); Nicolas Terzi (CHU de Grenoble Alpes, Department of Medical Intensive Care, F-38000 Grenoble, France and INSERM, U1042, University of Grenoble-Alpes, HP2, F-38000 Grenoble, France); Pascal Hazera and Michel Ramakers (Department of Intensive Care Medicine, General Hospital, Saint Lô, France); Djillali Annane and Andréa Polito (Service de Médecine Intensive et Réanimation, Hôpital Raymond Poincaré (APHP) and Laboratoire Infection & Inflammation, U1173 Université de Versailles SQY–Paris Saclay, INSERM General Intensive Care Unit, Raymond Poincaré Hospital, Garches, France U1173 Lab Inflammation & Infection, University of Versailles SQY-Paris Saclay - INSERM, Garches, France); Vincent Labbe and Muriel Fartoukh (Service de Médecine intensive Réanimation, AP-HP. Sorbonne University, Hôpital Tenon; Groupe de Recherche Clinique CARMAS, Collégium Gallilée); Bernard Floccard (Department of Anesthesiology and Critical Care Medicine, Edouard Herriot Hospital, Hospices Civils de Lyon, Lyon, France); Gérard Alvado and Olivier Cabon (Department of Intensive Care Medicine, General Hospital, Bayeux, France); Mehdi Bousta (Department of Intensive Care Medicine, General Hospital, Havre, France); Jean-Philippe Rigaud (Department of Intensive Care Medicine, General Hospital, Dieppe, France Department of Intensive Care, Dieppe General Hospital, Dieppe, France); and Claire Andrejak (Respiratory and Intensive Care Unit, University Hospital Amiens, Amiens, 80054, France).

**Table of contents**

1. **Supplemental information**

**1.1 Online Resource** 1. Detailed information regarding inclusion, no inclusion and exclusion criteria and definitions

1. **Supplemental Figure**

**1.2** **Online Resource 2.** PCT levels at inclusion (PCT-H_0_), at six hours (PCT-H_6_) and day 1 (PCT-H_24_) after inclusion, in subgroups of patients with (ATB^+^) (Panel a-c) and without (ATB^-^) (Panel d-f) antibiotics at inclusion.*****p<0.05 ***p<0.001

**1.3 Online Resource 3.** PCT levels at inclusion (PCT-H_0_), at six hours (PCT-H_6_) and day 1 (PCT-H_24_) after inclusion, in subgroups of patients with (PNP^+^) (Panel a-c) or without (PNP^-^) (Panel d-f) pneumonia at inclusion.*****p<0.05 **p<0.01

**1.4 Online Resource 4.** Receiver operating characteristic (ROC) curves at any time (i.e., H_0_, H_6_ and H_24_ after inclusion) for the prediction of documented bacterial infection (including bacterial and viral coinfection) vs nondocumented bacterial infection (i.e., documented viral infection alone or absence of documented pathogen) for the PCT levels in subgroups of patients with (ATB^+^) (Panel a-c) and without (ATB^-^) (Panel d-f) antibiotics at inclusion.

**1.5 Online Resource 5**. Receiver operating characteristic (ROC) curves at any time (i.e., H_0_, H_6_ and H_24_ after inclusion) for the prediction of documented bacterial infection (including bacterial and viral coinfection) vs nondocumented bacterial infection (i.e., documented viral infection alone or absence of documented pathogen) for the PCT levels in subgroups of patients with (PNP^+^) (Panel a-c) or without (PNP^-^) (Panel d-f) pneumonia at inclusion.

**Supplemental information**

**1.1 Online Resource 1**. Detailed information regarding inclusion, no inclusion and exclusion criteria and definitions

**Inclusion, no inclusion and exclusion criteria adult**

Patients experiencing severe AECOPDs with suspected lower respiratory tract infections with or without pneumonia who were admitted to the ICU were eligible. The time interval between hospital admission and inclusion in the study was required to be less than 48 hours.

Patients under 18 years, patients with known pregnancies, patients with clinical evidence of infection other that a lower respiratory tract infection, patients severe acute asthma, patients who were moribund or suffering from a disease with an estimated survival time of less than three months, patients who were severely immunosuppressed (i.e., patients with HIV infection, patients with neutropenia, stem cell transplant recipients, patients receiving immunosuppressive treatments, and patients receiving corticosteroid treatment at a dose greater than 0.5 mg / kg / day for more than 10 days), patients with nosocomial infections, patients who refused to participate in the study, patients who were included in another biomedical research protocol that was in progress or lasted less than 30 days were excluded from the study, and patients with a known PCT level at the time of their ICU admission.

**Definitions**

COPD was defined as a forced expiratory volume in 1 second/functional vital capacity (FEV1/FVC) ratio less than 70%, according to the 2005 Global Initiative for Chronic Obstructive Lung Disease Guidelines (GOLD) (<http://www.goldcopd.org>). COPD was classified as mild (FEV1 ≥80% of predicted), moderate (FEV1 ≥50% to <80%), severe (FEV1≥ 30% to <50%) and very severe (FEV1 <30%). An AECOPD was defined as a sustained worsening of the clinical condition of a patient with underlying COPD that results in a loss of stability, is beyond normal day-to-day variations, is acute in onset and necessitates a change in regular medications.

Pneumonia was defined as a new infiltrate on chest radiography accompanied by one or several of the following items: dyspnoea; cough; sputum production; fever above 38 °C; auscultatory findings, such as abnormal breath sounds and rales; leukocytosis (10,000 / mm³); or leukopenia (below 4000 / mm³).

We used the pneumonia severity index to estimate pneumonia severity.

AECOPD was attributed to a bacterial infection in cases in which the Gram stain results for the respiratory sample were positive; the pathogen concentration was greater than 10^5^ cfu/mL in a tracheobronchial aspiration or sputum sample, 10^4^ cfu/mL in a bronchoalveolar lavage fluid sample or 10^3^ cfu/mL in a distal protected specimen; a blood culture was found to be positive for a bacterial pathogen in the absence of an extrapulmonary focus of infection; a urine sample was found to be positive for Legionella pneumophila serogroup 1 antigen or antibodies to Legionella pneumophila; and a urine sample was found to be positive for pneumococcus antigen.

A culture, indirect immunofluorescence assay (IFA) or polymerase chain reaction (PCR) assay positive for viruses was considered evidence that the AECOPD was caused by a viral pathogen.

**Supplemental Figure**

**1.2 Online Resource 2.** PCT levels at inclusion (PCT-H_0_), at six hours (PCT-H_6_) and day 1 (PCT-H_24_) after inclusion, in subgroups of patients with (ATB^+^) (Panel a-c) and without (ATB^-^) (Panel d-f) antibiotics at inclusion.

*****p<0.05 ***p<0.001


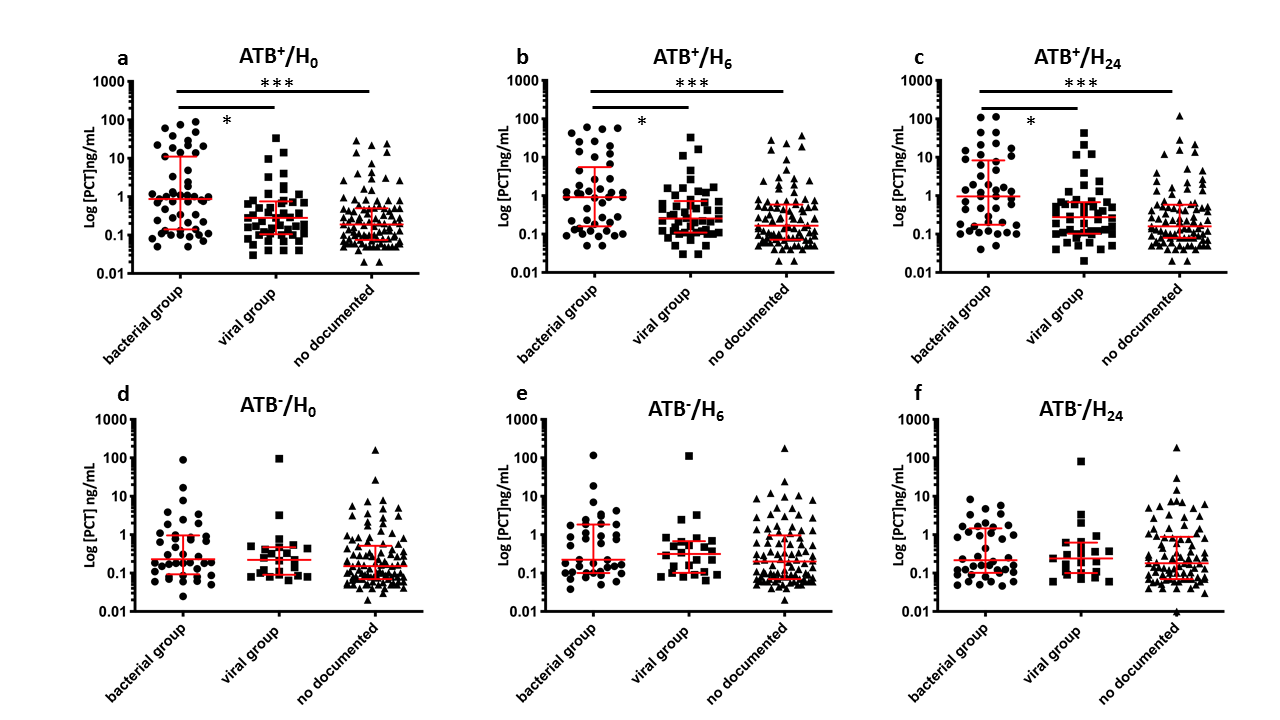


***Comment on Online Resource 2.***

In the subgroup of patients with antibiotics, PCT levels significantly differed between groups. PCT levels were significantly higher in patients with documented bacterial infection compared to the other groups. No difference in PCT levels was observed in the subgroup of patients without antibiotics at the time of inclusion

**1.3 Online Resource 3.** PCT levels at inclusion (PCT-H_0_), at six hours (PCT-H_6_) and day 1 (PCT-H_24_) after inclusion, in subgroups of patients with (PNP^+^) (Panel a-c) or without (PNP^-^) (Panel d-f) pneumonia at inclusion.

*****p<0.05 **p<0.01

**
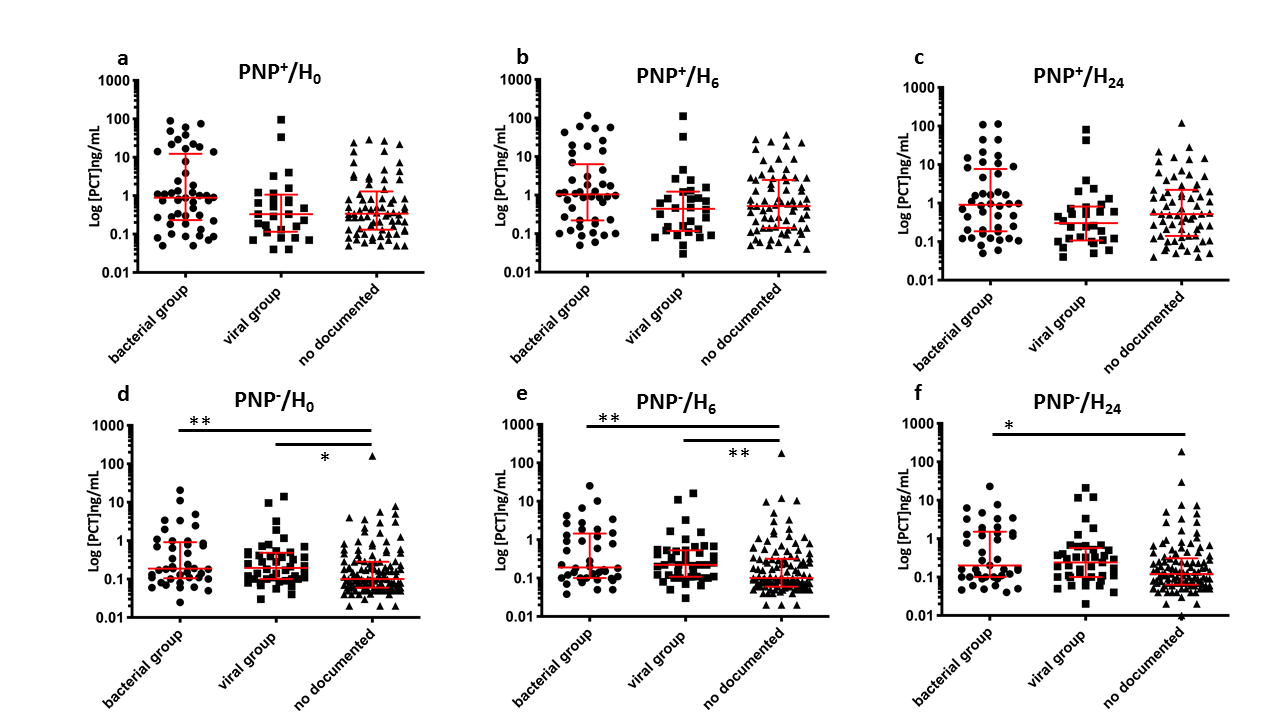
**

***Comment on Online Resource 3.***

In the subgroup with pneumonia, we observed no difference in PCT levels between the different groups. In contrast, in the subgroup of patients without pneumonia, PCT levels significantly differed between groups. PCT levels were significantly higher in patients with documented infection (i.e., bacterial and viral groups) compared to patients without documented pathogen.

**1.4 Online Resource 4.** Receiver operating characteristic (ROC) curves at any time (i.e., H_0_, H_6_ and H_24_ after inclusion) for the prediction of documented bacterial infection (including bacterial and viral coinfection) vs nondocumented bacterial infection (i.e., documented viral infection alone or absence of documented pathogen) for the PCT levels in subgroups of patients with (ATB^+^) (Panel a-c) and without (ATB^-^) (Panel d-f) antibiotics at inclusion.


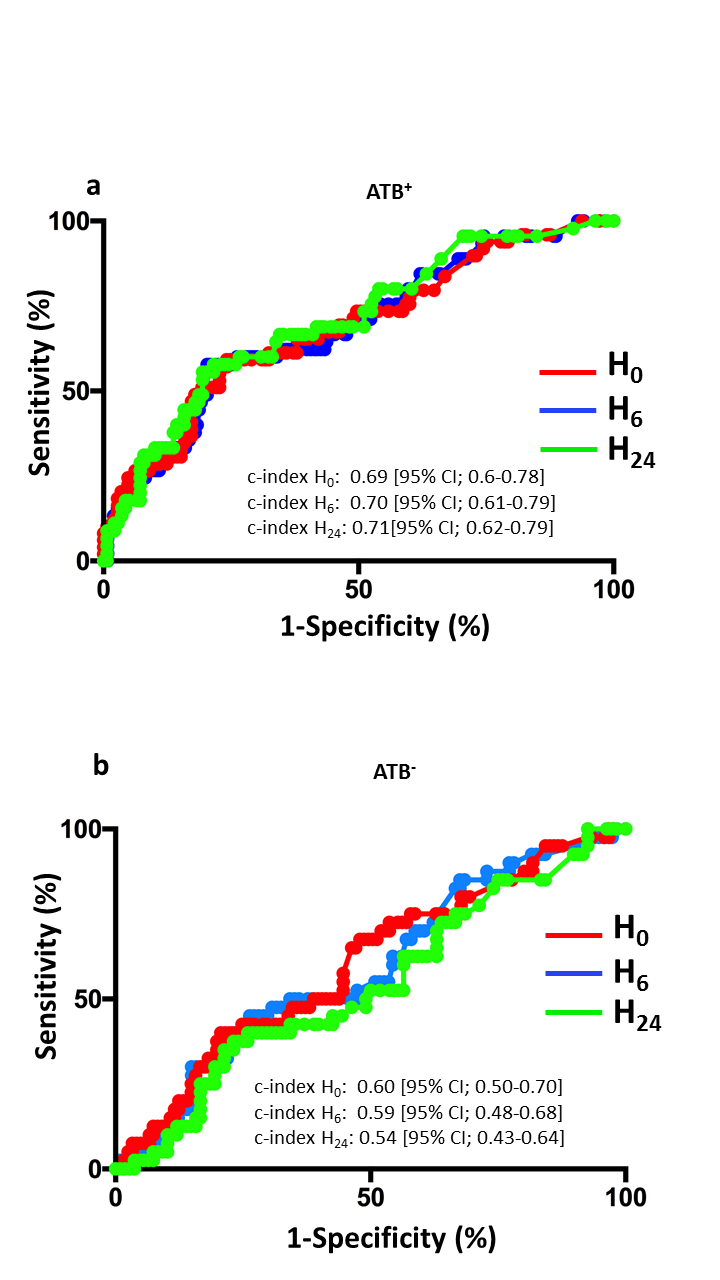


**1.5 Online Resource 5**. Receiver operating characteristic (ROC) curves at any time (i.e., H_0_, H_6_ and H_24_ after inclusion) for the prediction of documented bacterial infection (including bacterial and viral coinfection) vs nondocumented bacterial infection (i.e., documented viral infection alone or absence of documented pathogen) for the PCT levels in subgroups of patients with (PNP^+^) (Panel a-c) or without (PNP^-^) (Panel d-f) pneumonia at inclusion.


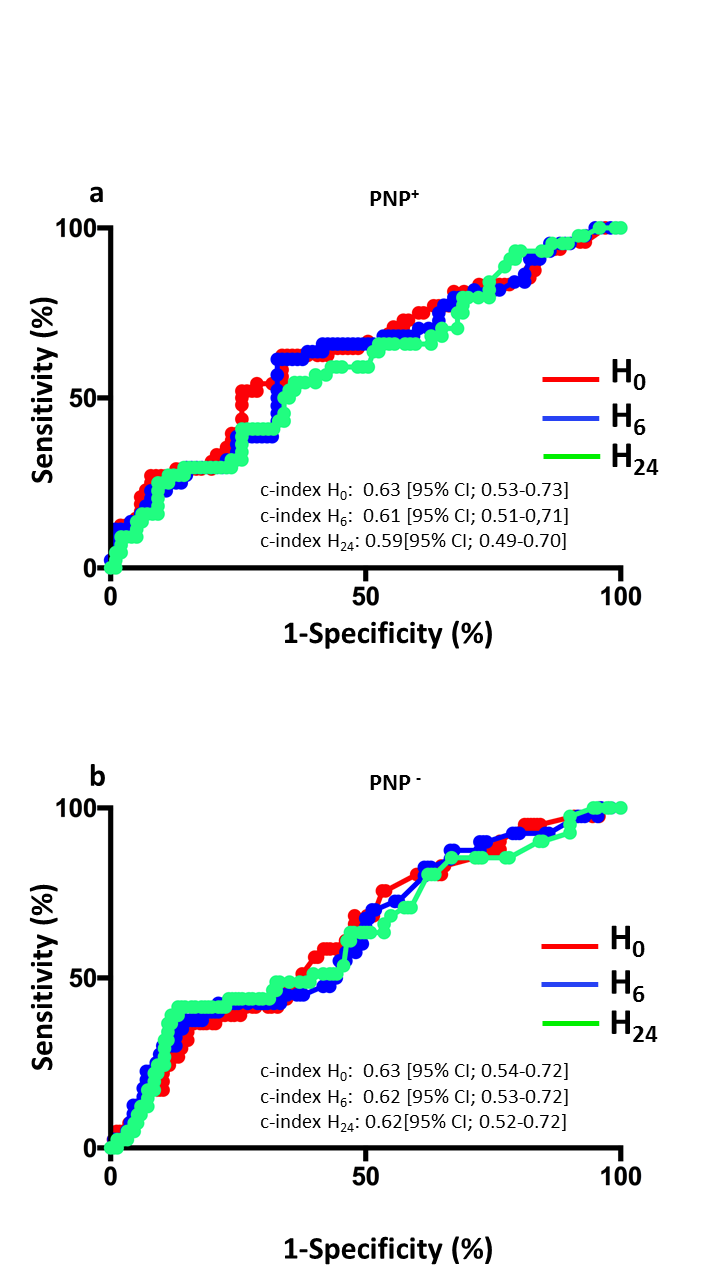

Supplement: Supplementary file 1 — Additional file 1: Online Resource 1. Detailed information regarding inclusion, no inclusion and exclusion criteria and definitions. Online Resource 2. PCT levels at inclusion (PCT-H0), at six hours (PCT-H6) and day 1 (PCT-H24) after inclusion, in subgroups of patients with (ATB+) (Panel a-c) and without (ATB-) (Panel d-f) antibiotics at inclusion. Online Resource 3. PCT levels at inclusion (PCT-H0), at six hours (PCT-H6) and day 1 (PCT-H24) after inclusion, in subgroups of patients with (PNP+) (Panel a-c) or without (PNP-) (Panel d-f) pneumonia at inclusion. Online Resource 4. Receiver operating characteristic (ROC) curves at any time (i.e., H0, H6 and H24 after inclusion) for the prediction of documented bacterial infection (including bacterial and viral coinfection) vs nondocumented bacterial infection (i.e., documented viral infection alone or absence of documented pathogen) for the PCT levels in subgroups of patients with (ATB+) (Panel a-c) and without (ATB-) (Panel d-f) antibiotics at inclusion. Online Resource 5. Receiver operating characteristic (ROC) curves at any time (i.e., H0, H6 and H24 after inclusion) for the prediction of documented bacterial infection (including bacterial and viral coinfection) vs nondocumented bacterial infection (i.e., documented viral infection alone or absence of documented pathogen) for the PCT levels in subgroups of patients with (PNP+) (Panel a-c) or without (PNP-) (Panel d-f) pneumonia at inclusion. [file 13613_2021_816_MOESM1_ESM.docx]
